# End-to-End PET/CT Interpretation and Quantification with an LLM-Orchestrated AI Agent: A Real-World Pilot Study

**DOI:** 10.2967/jnumed.126.272362

**Published:** 2026-10

**Authors:** Hongyoon Choi, Sungwoo Bae, Kwon Joong Na

**Affiliations:** 1 Department of Nuclear Medicine, Seoul National University Hospital, Seoul, Republic of Korea;; 2 Department of Nuclear Medicine, Seoul National University College of Medicine, Seoul, Republic of Korea;; 3 Portrai, Seoul, Republic of Korea; and; 4 Department of Thoracic and Cardiovascular Surgery, Seoul National University Hospital, Seoul National University College of Medicine, Seoul, Republic of Korea

**Keywords:** PET/CT, large language model, AI agent, workflow automation, quantitative imaging

## Abstract

Although deep learning models have improved individual PET analysis, image processing, and quantification tasks, end-to-end automation from raw DICOM data to quantitative clinical reporting remains limited, particularly in heterogeneous real-world settings. **Methods:** As a proof-of-concept, an autonomous large language model (LLM)–orchestrated multitool agent for end-to-end PET/CT interpretation was developed. A reasoning-based, text-based LLM selected appropriate series from raw DICOM, coordinated registration and SUV conversion, invoked segmentation and detection tools, generated maximum-intensity projections, called a vision-enabled LLM for interpretation, and synthesized structured draft reports. The system was retrospectively evaluated in 170 patients undergoing baseline [^18^F]FDG PET/CT for lung cancer staging, with clinical reports serving as the reference standard. **Results:** The agent successfully completed the full end-to-end workflow from raw DICOM selection to structured draft report generation without human intervention in all 170 examinations. Primary tumor detection achieved 100% sensitivity. For nodal involvement, sensitivity was 84.8% and specificity was 39.4%, whereas distant metastasis detection showed 70.2% sensitivity and 65.0% specificity. Discrepancy analysis of 58 nodal and 57 metastatic mismatch cases revealed systematic false-positive findings related to reactive or physiologic uptake and false-negative findings involving small-volume or anatomically atypical metastases. **Conclusion:** LLM-orchestrated PET/CT agents enabled workflow-level automation from raw DICOM to quantification and structured draft reporting under real-world conditions. Although primary tumor detection was highly reliable, nodal and metastatic assessment revealed systematic limitations, supporting a collaborative role with continued expert oversight in complex clinical scenarios.

## Visual Abstract

**Figure:**
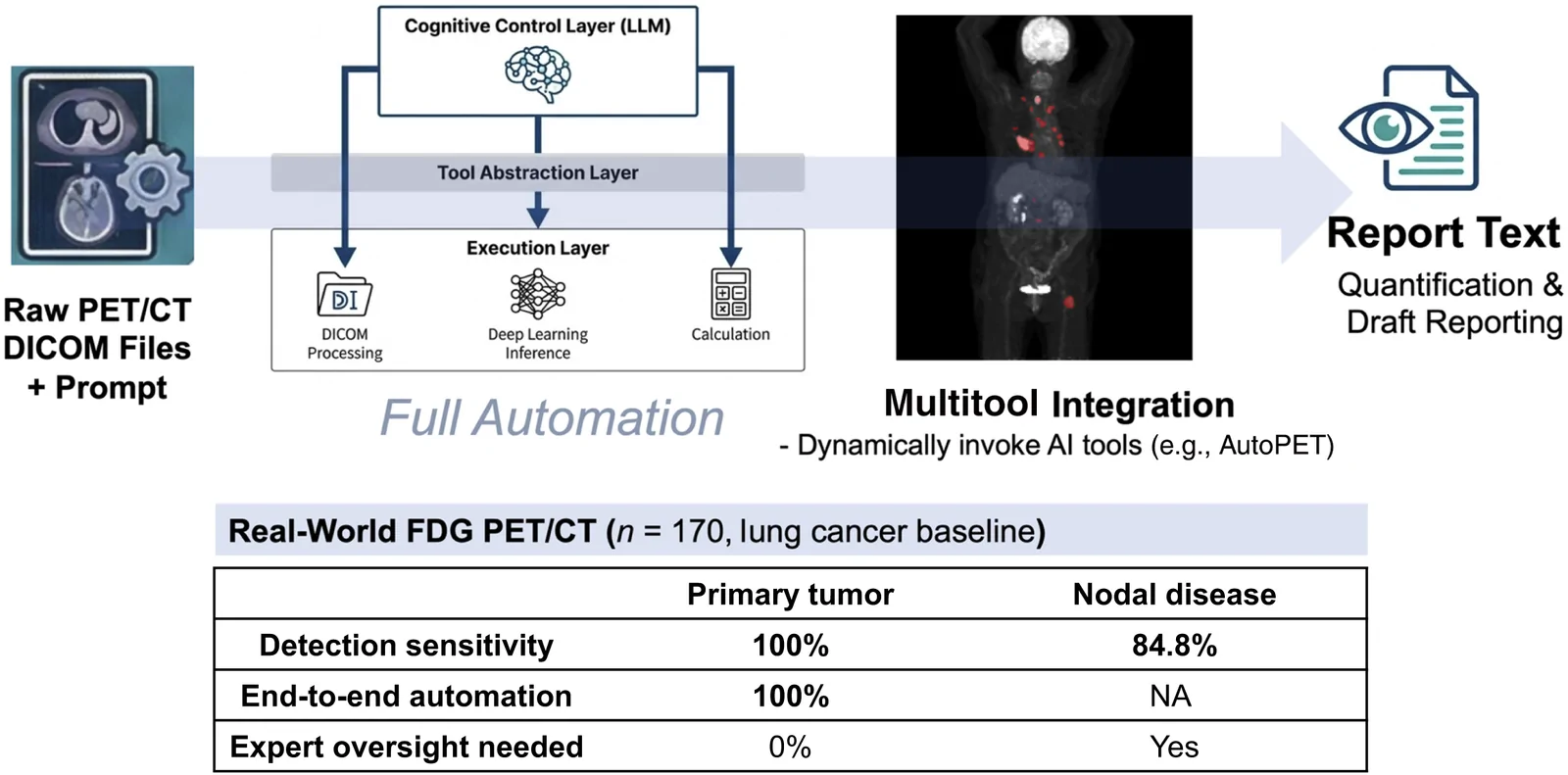

PET/CT provides both qualitative and quantitative information across a wide range of clinical applications, including SUVs and tumor burden metrics (1). Recent advances in task-specific artificial intelligence (AI) have enabled automated components of PET analysis (2,3), such as organ segmentation for regional quantification and lesion-focused measurements, including metabolic tumor volume (4). However, real-world clinical PET/CT interpretation requires a tightly coupled, multistep workflow that extends beyond isolated segmentation or quantification tasks (5). Routine clinical interpretation involves a quantitative imaging process that requires multiple image-processing steps, which may vary across centers and clinical scenarios. These steps include selecting appropriate image series from raw DICOM studies, performing registration and resampling, and converting raw counts to SUVs using patient and injection metadata. After this quantitative preprocessing, nuclear medicine physicians identify and segment relevant lesions (either semiautomatically or manually), assess whole-body disease burden, and synthesize structured reports tailored to the specific clinical question (5). Despite recent progress in AI tools, these workflow-level steps remain largely performed or supervised by expert readers (2,3).

This gap becomes particularly pronounced in real-world clinical environments, where PET/CT data are highly heterogeneous across scanners, acquisition protocols, reconstruction settings, SUV scaling, and metadata completeness. Such variability poses a major barrier to fully automated interpretation, as an effective system must robustly select appropriate series, resolve inconsistencies in DICOM information, and coordinate multiple downstream tools under uncertain conditions. However, most existing segmentation and detection approaches are developed using curated datasets with structured situations and do not address these workflow-level challenges (6–9). Multimodal large language models (LLMs) may help bridge this gap by reasoning over text, images, and metadata to orchestrate complex imaging pipelines (10,11), although their clinical deployment requires careful definition of appropriate roles and oversight (12,13).

In this study, we evaluated an autonomous, LLM-orchestrated multitool agent designed to automate end-to-end [^18^F]FDG PET/CT interpretation as a proof-of-concept study. Unlike conventional single-task models, the proposed system functions as a cognitive orchestrator that coordinates image processing, quantitative analysis, lesion detection, and structured report generation in a manner aligned with real-world clinical workflows (14,15). Guided by simple text prompts, the agent dynamically selects appropriate PET/CT series, invokes specialized tools, and generates quantitative outputs and draft reports interactively (14). We retrospectively validated the system in a cohort of 170 patients undergoing baseline lung cancer staging, assessing workflow-level robustness, diagnostic performance, and the clinical acceptability of generated reports. Our findings illustrate the potential role of general-purpose AI agents as workflow-level assistants that provide structured analytic baselines while maintaining the need for expert oversight in complex clinical scenarios (13,15).

## MATERIALS AND METHODS

### Study Design

This retrospective single-center study used clinical PET/CT imaging data obtained from the institutional clinical data warehouse. We identified patients who underwent routine [^18^F]FDG PET/CT for lung cancer staging in 2018 and extracted a cohort of 170 cases for pilot evaluation of the proposed workflow-based approach. In addition, publicly available PET datasets from The Cancer Imaging Archive (16) were used for application tests. Institutional review board approval was obtained (IRB 2105-157-1221), and the requirement for written informed consent was waived because of the retrospective design and the use of deidentified data.

### PET Dataset

The study cohort consisted of 170 adult patients who underwent baseline [^18^F]FDG PET/CT for the diagnostic work-up or initial staging of suspected or newly diagnosed primary lung cancer. Baseline staging scans were defined as the first PET/CT examination performed before any systemic or local oncologic treatment. Studies performed for restaging, treatment response assessment, or surveillance were excluded. Additional exclusion criteria included incomplete PET or CT series and corrupted DICOM files that could not be parsed. All examinations were acquired using routine clinical protocols on heterogeneous PET/CT systems. Variability in series naming conventions, reconstruction parameters, voxel sizes, and SUV scaling reflected real-world clinical conditions.

For the single-center cohort, patients fasted for at least 8 h, and blood glucose levels were measured before tracer injection. [^18^F]FDG was administered at a dose of 5.18 MBq/kg when blood glucose was below 200 mg/dL. After voiding, PET/CT imaging was performed approximately 60 min after injection. Scans were acquired using 3 dedicated PET/CT systems (Biograph TruePoint 40, Biograph mCT 40, and Biograph mCT 64; Siemens Healthineers). Low-dose non–contrast-enhanced CT was obtained from the skull base to the proximal thigh (120 kVp; matrix size, 512 × 512; slice thickness, 5 mm for TruePoint 40 and 3 mm for mCT systems). Subsequently, PET emission imaging was performed, with acquisition times of 2 min/bed (TruePoint) and 1 min/bed (mCT systems).

### LLM-Orchestrated Agent Architecture

The proposed system was designed using a 3-layer architecture consisting of a cognitive control layer, a tool abstraction layer, and an execution layer (10,14). The overall architecture and data flow from raw DICOM input to quantitative report generation are illustrated in Figure 1. The cognitive control layer was implemented using a text-based LLM (Gemini 3 Flash Preview; Google) (17), which functioned as the workflow orchestrator. A predefined system prompt specified its role as a PET/CT interpretation assistant responsible for planning the analysis, selecting appropriate tools, monitoring intermediate outputs, and generating structured draft reports. Task-specific prompt templates guided key steps, including DICOM series selection, image preprocessing, lesion quantification, and SUV metric computation. Tool interactions were performed through a structured schema defining tool names, required inputs, and expected outputs. The orchestrator used an iterative reasoning framework in which each tool call followed a thought–action–observation sequence, enabling stepwise planning and validation (18). All reasoning steps and tool invocations were logged. The tool abstraction layer translated high-level requests from the orchestrator (e.g., lesion segmentation, background SUV calculation) into executable functions. Tool metadata defined input modalities (PET, CT, masks, or derived images), file formats, and expected behavior, allowing the system to detect failures or empty outputs and adapt accordingly. The execution layer consisted of Python-based modules responsible for DICOM handling, image registration and resampling, SUV computation, visualization, and deep learning–based segmentation and detection.

**FIGURE 1.**
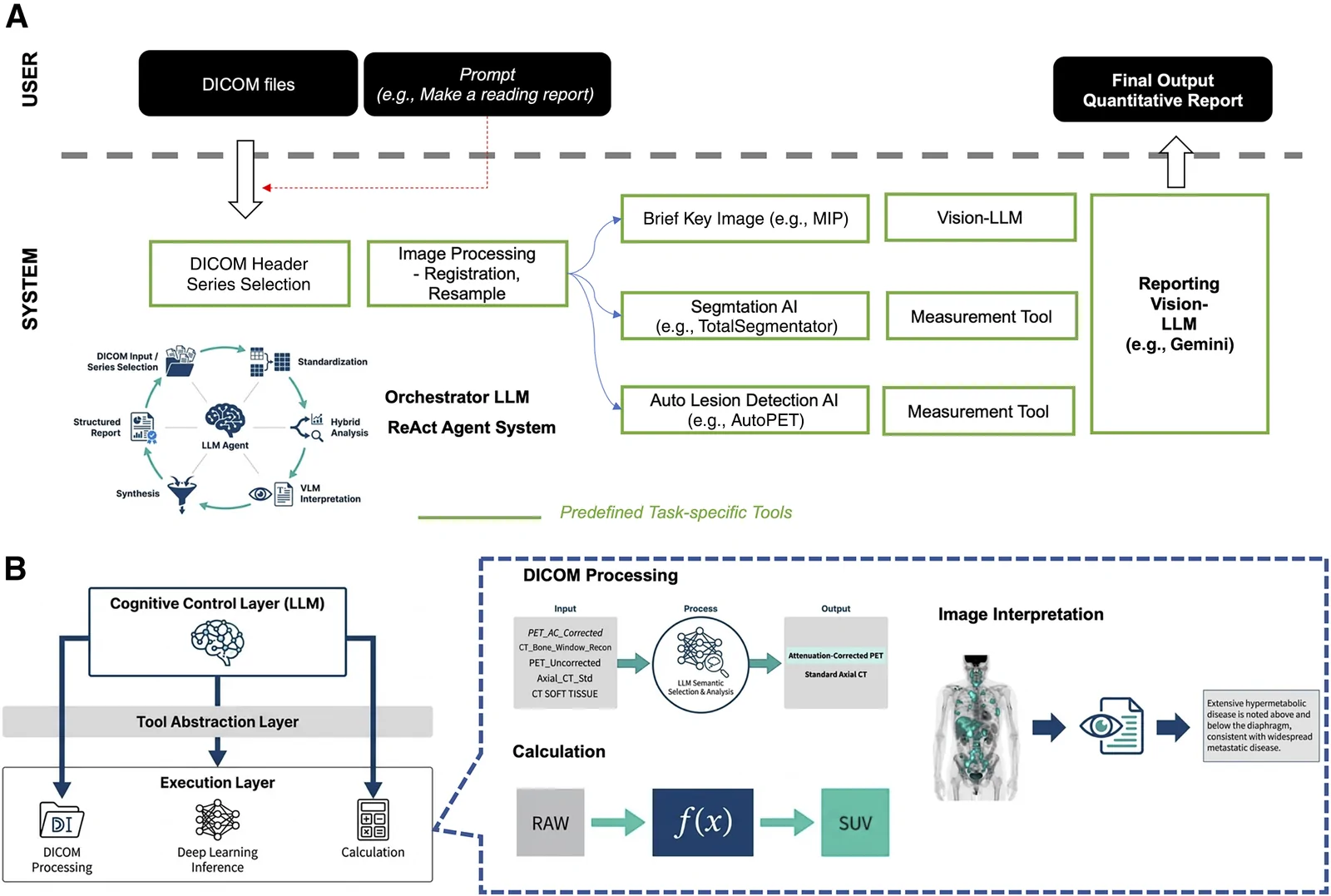
Architecture of LLM-orchestrated multitool PET/CT workflow. Schematic overview of proposed LLM-orchestrated agent for end-to-end PET/CT interpretation and quantitative reporting. (A) Workflow from raw DICOM files to structured report generation. LLM-based cognitive control layer performs series selection, coordinates preprocessing (e.g., registration, resampling), and dynamically invokes task-specific tools for image generation, segmentation, detection, and quantification. Outputs are integrated and passed to vision-enabled LLM (Vision-LLM) to generate structured report. (B) Three-layer architecture consisting of cognitive control layer (planning and orchestration), tool abstraction layer (mapping tasks to modules), and execution layer (DICOM data processing, model inference, and quantification). MIP = maximum-intensity projection.

### Imaging Tools, Quantification, and Workflow Execution

The agent integrated multiple task-specific tools to enable end-to-end PET/CT interpretation and quantification. The primary aim of this study was not to develop new models for individual tasks, such as lesion segmentation, organ delineation, or quantitative measurement. Rather, the goal was to evaluate a workflow-level agent capable of interactively orchestrating existing specialized tools in response to user prompts, more closely resembling routine clinical PET/CT interpretation processes.

Whole-body PET lesion segmentation was performed using an AutoPET nnU-Net–based 3-dimensional model trained for volumetric tumor delineation (7–10). Resulting masks were used to compute quantitative metrics, including SUV_max_, metabolic tumor volume, and total lesion glycolysis (4). Organ segmentation was performed on CT using TotalSegmentator to obtain major anatomic structures and reference regions (19).

A vision-enabled LLM (Gemini 3 Flash Preview) was used for image-based interpretation (10,17). The orchestrator generated standardized key visualizations, including whole-body PET maximum-intensity-projection images, multiplanar PET/CT fusion slices, and lesion or organ mask overlays, and prompted the vision-enabled LLM to provide lesion-level summaries of location, uptake intensity, and suspected category (primary, nodal, metastatic, or physiologic). These outputs were incorporated into staging assessment and structured report generation.

For each study, the orchestrator parsed raw DICOM series and selected an attenuation-corrected whole-body PET series with the corresponding CT. PET and CT volumes were aligned and resampled into a common space to ensure consistent fusion and quantification. SUVs were computed voxelwise using injected activity, patient weight, and acquisition timing extracted from DICOM metadata (1). Lesion- and organ-level quantitative summaries were then automatically generated and synthesized into staging-oriented draft reports (5).

Tool calls were monitored using predefined error-detection rules, including checks for missing outputs, invalid quantitative ranges, or run time failures. When lesion segmentation failed or yielded empty masks, the system applied fallback strategies, such as relying on the vision-enabled LLM interpretation of maximum-intensity-projection and fusion images for staging decisions. All intermediate reasoning steps, tool inputs and outputs, and error events were logged. A subset of cases was selected for qualitative analysis of the agent’s workflow traces.

### Mapping to Staging Labels and Reference Standard

For each patient, the original clinical PET/CT report served as the reference standard. Reports were reviewed to extract patient-level labels for primary tumor presence, nodal involvement (node positive vs. node negative), and distant metastasis (presence or absence). Agent outputs were mapped to the same categories using predefined operational criteria. Primary tumor presence was defined as at least 1 [^18^F]FDG-avid lesion within the lung parenchyma or hilum classified as primary. Nodal involvement was defined as any lesion categorized as nodal disease in mediastinal, hilar, supraclavicular, or other nodal stations. Distant metastasis was defined as [^18^F]FDG-avid lesions outside the thoracic nodal basin labeled as metastatic, including involvement of bone, the liver, adrenal glands, the brain, or other organs. Discrepancies between agent-derived and reference labels were recorded and subsequently categorized as false-positive or false-negative patterns for nodal involvement and metastatic disease.

### Statistical Analysis

Workflow-level feasibility was defined as the proportion of examinations in which the agent completed the full end-to-end pipeline from DICOM input to draft report generation without human intervention. Patient-level diagnostic performance for primary tumor detection, nodal involvement, and distant metastasis was evaluated using sensitivity, specificity, accuracy, positive predictive value, and negative predictive value, with the clinical report serving as the reference standard. Confusion matrices were generated for each task.

## RESULTS

### Reactive Orchestrator-Based Outputs

The agent was tested across multiple clinically relevant prompt-driven scenarios, including qualitative draft reporting, SUV measurement, and quantitative lesion reporting. For qualitative interpretation, the prompt “Interpret the PET scan and draft a report for any abnormalities” triggered the agent to generate whole-body maximum-intensity-projection images and request vision-enabled LLM–based assessment of abnormal [^18^F]FDG uptake patterns (Fig. 2A).

**FIGURE 2.**
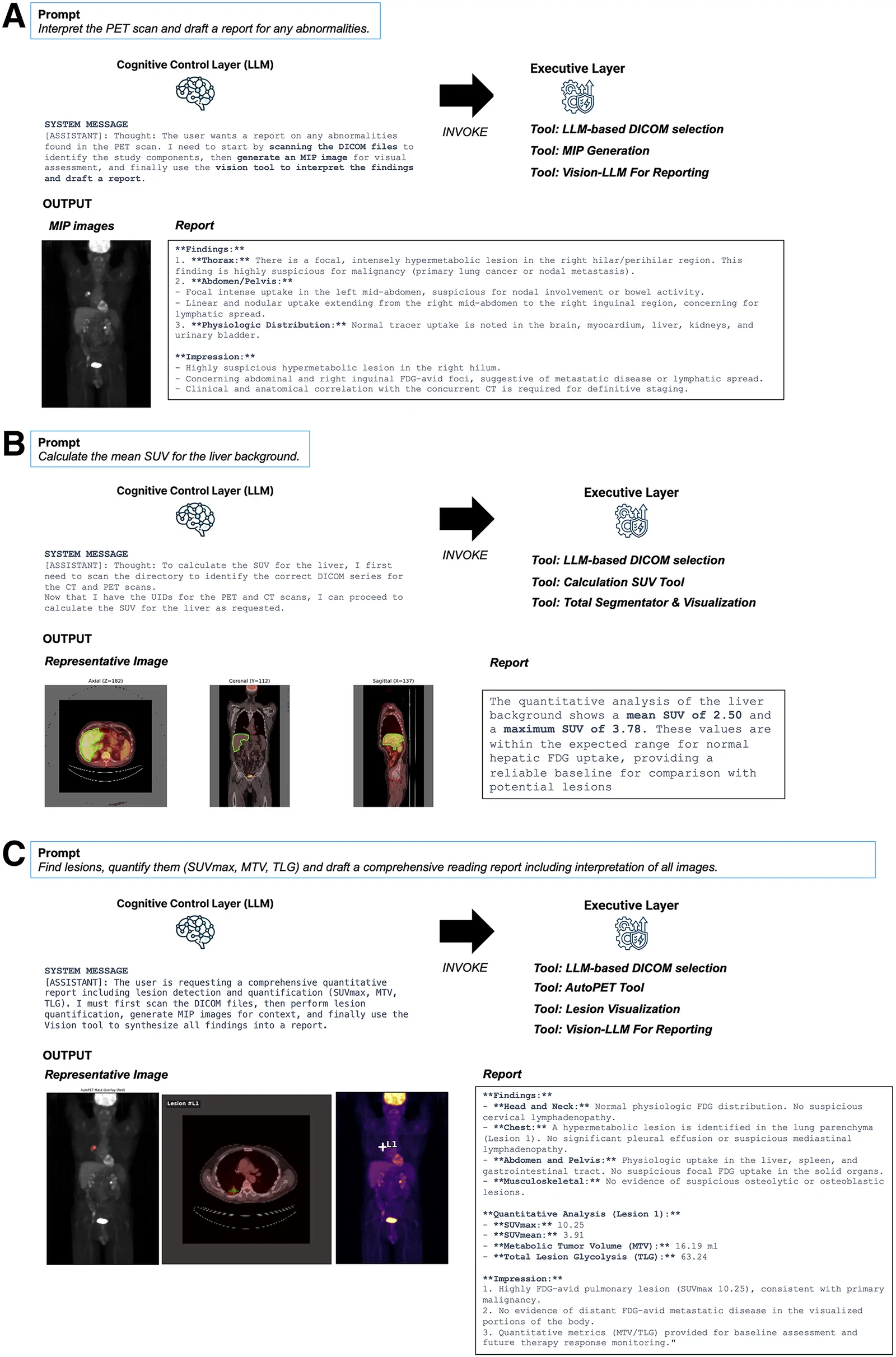
Prompt-driven LLM-agent orchestration for qualitative interpretation and quantitative PET/CT analysis. (A) Qualitative draft reporting. Given prompts for interpretation, LLM selects appropriate PET/CT series, generates whole-body maximum-intensity-projection (MIP) images, and invokes vision-enabled LLM (Vision-LLM) to produce structured report of abnormal [^18^F]FDG uptake. (B) Organ-based quantitative SUV computation. For liver SUV estimation, agent performs CT-based organ segmentation (TotalSegmentator) and computes SUV metrics, providing quantitative summaries with representative fusion images. (C) End-to-end lesion detection and reporting. For comprehensive analysis, agent coordinates AutoPET-based segmentation, visualization, and quantitative measurements (SUV_max_, metabolic tumor volume [MTV], total lesion glycolysis [TLG]), followed by vision-enabled LLM–assisted structured reporting.

For quantitative tasks, the agent dynamically invoked task-specific imaging tools, including TotalSegmentator for organ delineation (19) (Fig. 2B) and an AutoPET lesion segmentation model, for tumor quantification on the basis of the prompts (6–8). Using these outputs, the system automatically computed standard PET metrics, such as lesion SUV and metabolic tumor volume (Fig. 2C). An example of the agent’s interactive execution, including its reasoning process and tool invocation guided by system prompts, is provided in Supplemental Video 1 (supplemental materials are available at http://jnm.snmjournals.org).

End-to-end execution of the predefined workflow—from DICOM loading and series selection through registration/resampling, SUV conversion, segmentation, vision-enabled LLM consultation, and structured draft report generation—was successfully completed in most examinations. Failures were rare (1 in 170 cases) and were primarily attributable to DICOM or metadata inconsistencies, such as insufficient information for SUV calculation. In cases where quantitative lesion tools failed, the agent reverted to a qualitative interpretation mode using maximum-intensity-projection–based vision-enabled LLM assessment to generate clinically meaningful draft reports (Supplemental Fig. 1).

### Patient-Level Staging Performance

Using expert-interpreted real clinical PET/CT reports as the reference standard, the agent’s staging outputs were evaluated at the patient level for primary tumor detection, nodal involvement, and distant metastasis. An example case is presented in Figure 3A, illustrating lesion detection and interpretation through an end-to-end pipeline, from raw DICOM images to the generated narrative report. Confusion matrices and summary performance metrics for these staging tasks are provided in Figure 3B.

**FIGURE 3.**
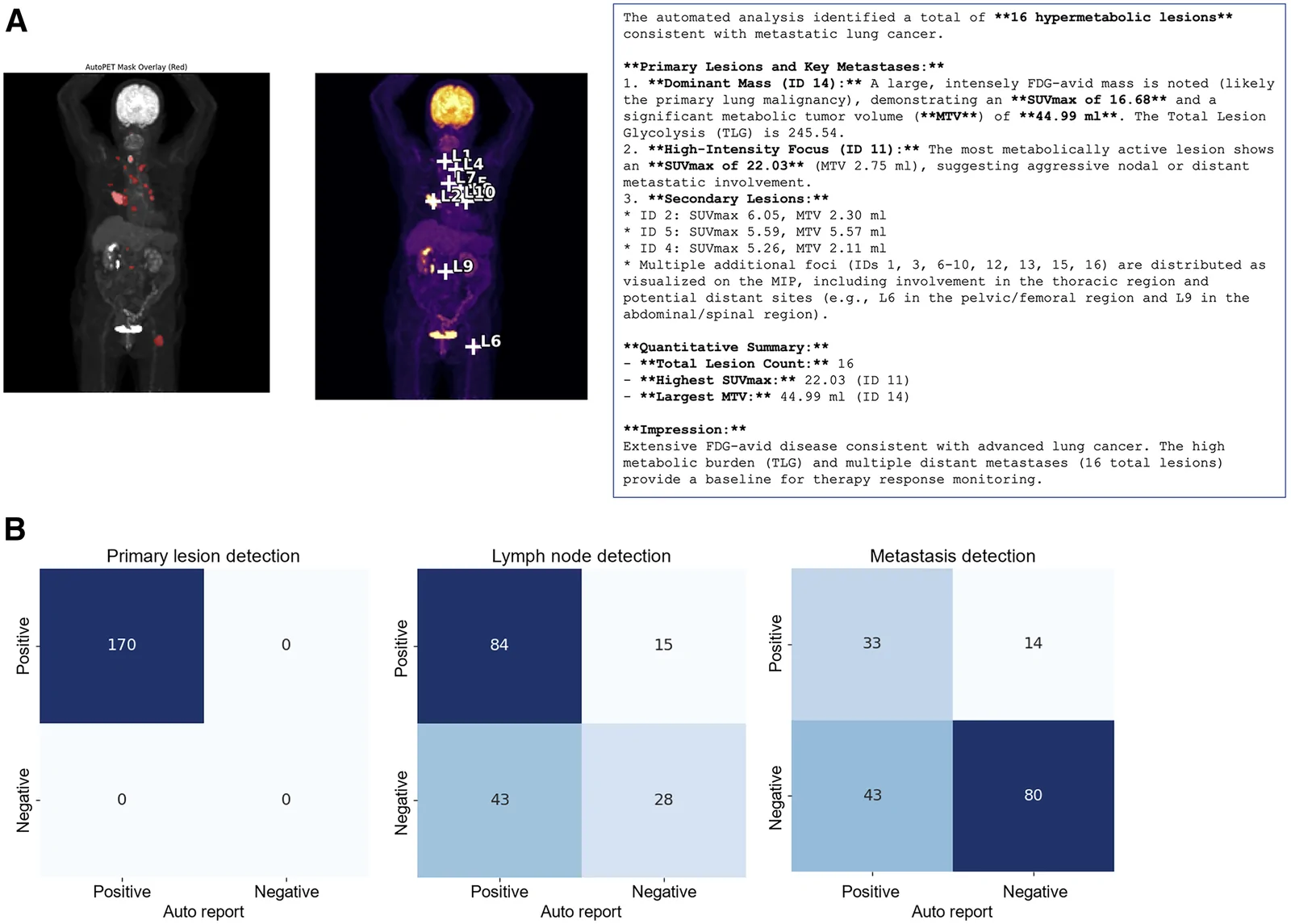
Representative lesion detection outputs and patient-level staging performance. (A) Examples of automated whole-body lesion detection and visualization. AutoPET-based segmentation masks (red overlays) projected on whole-body PET images, demonstrating detected [^18^F]FDG-avid lesions. Representative whole-body PET maximum-intensity-projection images with automatically labeled lesion indices (L1–L10), illustrating multilesion identification and spatial distribution. (B) Patient-level confusion matrices comparing agent-generated staging outputs with expert-interpreted clinical PET/CT reports for primary tumor detection, nodal involvement (node positive vs. node negative), and distant metastasis (presence vs. absence). For primary tumor detection, agent achieved 100% sensitivity (170/170). For nodal staging, sensitivity was 84.8% (84/99) and specificity was 39.4% (28/71). For distant metastasis detection, sensitivity was 70.2% (33/47) and specificity was 65.0% (80/123).

Patient-level staging performance was assessed against expert clinical PET/CT reports (Fig. 3). For primary tumor detection, the agent identified all primary lung cancers correctly, achieving a sensitivity of 100% (170/170). For nodal staging, the agent demonstrated high sensitivity for clinically significant lymph node involvement (84.8%, 84/99) but limited specificity (39.4%, 28/71), reflecting frequent false-positive nodal calls. Discrepancy analysis showed that most false positives arose from low-grade [^18^F]FDG uptake in hilar or mediastinal lymph nodes that were interpreted as reactive or physiologic in ground-truth expert reports. False-negative nodal cases were less common and typically involved small or mildly avid interlobar, paratracheal, or supraclavicular nodes that were omitted from the automated report. For distant metastasis detection, the agent achieved moderate sensitivity (70.2%, 33/47) and specificity (65.0%, 80/123), with an overall accuracy of 66.5%. Among the metastatic mismatches, false-positive classifications were frequently driven by physiologic uptake in the bowel or pelvis and benign osseous uptake related to degenerative or traumatic changes. In contrast, false negatives most often involved small-volume metastases at anatomically challenging or atypical sites, including subtle bone lesions, pleural seeding, adrenal involvement, thyroid nodules, or intracranial metastases. All false-positive and false-negative cases identified in the discrepancy analysis are summarized in the supplemental materials. A site-specific analysis of adrenal and brain metastases is summarized in Supplemental Figure 2. The agent showed low sensitivity for adrenal metastases (18.2%, 2/11) and did not detect brain metastases (0/3), whereas maintaining high specificity for both sites. Together, these discrepancy patterns indicate that the agent provides clinically meaningful staging support but remains vulnerable to borderline nodal uptake and physiologic mimics, underscoring the need for expert oversight in high-risk scenarios.

## DISCUSSION

This study demonstrated the feasibility of an LLM-orchestrated multitool agent capable of executing an end-to-end PET/CT workflow directly from heterogeneous clinical data. Unlike prior applications of LLMs in nuclear medicine, which primarily operate on preexisting reports or structured outputs (20,21), the present system coordinates image processing, quantitative computation, segmentation, and vision-based interpretation within a unified workflow (22). In a cohort of 170 baseline examinations for lung cancer staging, the agent completed the full pipeline, supporting the feasibility of workflow-level automation under real-world conditions (14,15).

A key contribution is the shift from isolated task performance to orchestration across the entire PET/CT interpretation process. Clinical PET reading requires coordinated steps including series selection, registration, SUV conversion, segmentation, and reporting. The LLM-based control layer enabled dynamic tool selection and validation, including fallback strategies when segmentation or metadata-dependent SUV computation failed. This architecture mirrors how human readers adapt to incomplete or inconsistent data and supports the role of LLMs as supervisory “control layers” rather than standalone diagnostic models (10,14). Methodologically, this study extends AI agents from text-based augmentation to coordinated image-based workflows. By integrating AutoPET segmentation and CT-based organ masks within a unified framework (8,9,19), we demonstrate how existing algorithms can be combined into a clinically applicable, quantification-ready pipeline without retraining. This approach supports scalable integration of validated tools while preserving expert oversight (13,15).

Diagnostic performance varied across staging components and was influenced in part by the capabilities of the final vision-enabled LLM responsible for image interpretation and report generation. Primary tumor detection achieved 100% sensitivity, reflecting robust identification of dominant lesions (23). In contrast, nodal and distant metastasis assessment showed moderate performance with asymmetric errors. Nodal staging showed high sensitivity but low specificity, mainly attributable to overcalling low-grade uptake in reactive lymph nodes. For distant metastases, false positives were often driven by physiologic or benign uptake, whereas false negatives involved small or atypical lesions. These patterns reflect known limitations of PET/CT interpretation in heterogeneous or borderline uptake scenarios (6,8,24). In this regard, the goal of the current AI agent is not to replace human expertise in PET/CT interpretation. Rather, such systems should be viewed as workflow-level assistants that can support physicians and researchers by automating repetitive preprocessing and quantitative tasks, providing preliminary lesion measurements and enabling scalable analysis across large and heterogeneous imaging cohorts.

Importantly, the agent’s behavior illustrates that orchestration does not eliminate the intrinsic strengths and weaknesses of underlying task-specific models. Instead, the LLM layer integrates their outputs and can mitigate some failures through reasoning and fallback strategies, but it cannot fully compensate for domain-generalization limitations or ambiguous uptake patterns (6–8). The system functions most appropriately as a workflow assistant rather than an autonomous reader. In particular, the current model showed limitations in detecting challenging lesions, such as adrenal and brain metastases. Sensitivity for adrenal metastases was low (2/11), and no brain metastases were detected. These findings likely reflect difficulties in small or complex lesions and the intrinsic limitations of [^18^F]FDG PET/CT for intracranial disease because of high physiologic uptake. Accordingly, the agent should be considered a workflow-level assistant rather than a standalone staging tool, particularly for clinically critical metastatic sites requiring expert review.

One important point is that the primary objective of this study was not to develop or benchmark the best-performing vision-enabled LLMs for interpretation. Rather, the main contribution was to demonstrate the feasibility of an end-to-end workflow-level agent that can autonomously coordinate quantitative and qualitative PET/CT interpretation steps under heterogeneous real-world clinical conditions. A preliminary comparison of the lung cancer cohort with 170 patients across different LLMs (Claude Sonnet 4.6 [Anthropic] and Gemma 4 26B [Google]) showed broadly similar performance patterns, suggesting that the overall framework is relatively robust (Supplemental Fig. 3). The interpretive performance of the system is partly dependent on the underlying vision-enabled LLM component, and future integration with emerging specialized medical vision–language foundation models may further improve diagnostic robustness and lesion characterization (e.g., MedGemma [Google], HuLu-Med) (25,26). Notably, such improvements could be achieved by substituting or upgrading the vision-enabled LLM module within the same orchestration architecture, allowing the agent to remain interactive and adaptable across diverse clinical scenarios and quantification purposes.

In addition, the proposed framework demonstrated robustness to variability across multiscanner data with differing DICOM structures, successfully completing end-to-end workflows. This reflects a key property of the LLM-based orchestration layer, which adaptively handles variations in metadata and preprocessing from DICOM selection to downstream analysis. In failure cases, the agent applied fallback strategies from a disrupted DICOM metadata to retain meaningful outputs (Supplemental Fig. 1). These results suggest practical resilience to domain shifts. Future work should incorporate explicit uncertainty estimation and systematic evaluation across unseen imaging protocols and institutions.

Several limitations must be acknowledged. The study is retrospective, and quantitative analysis was restricted to baseline [^18^F]FDG PET/CT for cancer staging. The reference standard in this study was derived from routine clinical PET/CT reports, reflecting the practical goal of evaluating the agent’s performance within an end-to-end real-world interpretation framework. System performance remains dependent on the domain generalizability of the underlying segmentation models and on the completeness and consistency of DICOM metadata, which continues to represent a major barrier to fully automated clinical pipelines (6,8). In addition, the clinical impact on reading time, decision-making, and patient outcomes was not evaluated. Future work should validate workflow-level agents across multiple institutions, tracers, and disease indications and should explore integration with next-generation multimodal models capable of deeper spatial reasoning (27,28). Standardized benchmarks for end-to-end imaging agents, complementing existing segmentation challenges (6,8), will be necessary to rigorously assess robustness under heterogeneous clinical conditions. In addition, the potential risk of hallucination in LLM-based systems should be considered. In this study, the agent was grounded through tool-based orchestration, where outputs were constrained by intermediate imaging results and structured workflows, with fallback mechanisms to limit error propagation. Nevertheless, the integration of explicit grounding strategies, such as retrieval-augmented generation or constrained prompting, will be important to enhance clinical reliability.

## CONCLUSION

An LLM-orchestrated multitool agent can automate most components of the [^18^F]FDG PET/CT staging workflow in real-world heterogeneous data. Although primary tumor detection approached expert-level sensitivity, nodal and metastatic assessment revealed characteristic, interpretable error patterns. These findings support the role of general-purpose AI agents as structured workflow assistants that enhance quantitative consistency and draft reporting while maintaining essential expert oversight in complex clinical scenarios.

## DISCLOSURE

This research was supported by Korea Medical Device Development Fund grant funded by the Korean government (the Ministry of Science and ICT, the Ministry of Trade, Industry and Energy, the Ministry of Health and Welfare, the Ministry of Food and Drug Safety) (project 1711137868, RS-2020-KD000006) and the NAVER Digital Bio Innovation Research Fund funded by NAVER Corporation (grant 3720230020). Hongyoon Choi and Kwon Joong Na are cofounders of Portrai. Kwon Joong Na receives a research grant from Inocras (USA). ChatGPT and Gemini were used exclusively for grammatical corrections and enhancements in clarity. No other potential conflict of interest relevant to this article was reported.

## Notes

KEY POINTS **QUESTION:** Can an LLM-orchestrated multitool agent automate the end-to-end [^18^F]FDG PET/CT reading workflow directly from heterogeneous clinical DICOM data? **PERTINENT FINDINGS:** The LLM-orchestrated agent dynamically generated qualitative reports and quantitative PET metrics from raw DICOM data in response to user prompts. In 170 baseline lung cancer PET/CT studies, the full workflow was completed without human intervention. Primary tumor detection achieved 100% sensitivity, whereas nodal involvement and distant metastasis assessment showed moderate performance with characteristic false-positive and false-negative patterns. **IMPLICATIONS FOR PATIENT CARE:** LLM-based workflow agents may provide proof-of-concept nuclear medicine imaging workflow-level support by dynamically coordinating multiple imaging tools for preprocessing, quantification, and draft reporting, while maintaining the need for expert oversight in complex clinical interpretation scenarios.
